# The DrinksRation Smartphone App for Modifying Alcohol Use Behaviors in UK Military Service Personnel at Risk of Alcohol-Related Harm: Protocol for a Randomized Controlled Trial

**DOI:** 10.2196/49918

**Published:** 2023-10-13

**Authors:** Kate King, Daniel Leightley, Neil Greenberg, Nicola Fear

**Affiliations:** 1 Academic Department of Military General Practice Research & Clinical Innovation Defence Medical Services Birmingham United Kingdom; 2 King's Centre for Military Health Research Institute of Psychiatry, Psychology and Neuroscience King's College London London United Kingdom

**Keywords:** alcohol drinking, alcohol brief intervention, digital intervention, military

## Abstract

**Background:**

Consumption of alcohol is synonymous with military populations, and studies have shown that serving personnel drink more than age- and sex-matched civilian populations. While ingrained in the military culture, excessive alcohol use is associated with increased rates of disciplinary issues, sickness absence, and loss of productivity, as well as contributing to a burden of acute and chronic health problems. Alcohol brief interventions can reduce alcohol use in civilian populations, but there is a paucity of evidence relating to the effectiveness of similar interventions in military populations. The DrinksRation smartphone app was designed to have a basis in behavior change technique theory and focuses on providing interactive behavioral prompts tailored to a military population. It has previously been shown to be effective in a help-seeking veteran population.

**Objective:**

The primary aim of the Military DrinksRation randomized controlled trial study is to determine whether it is similarly effective in a serving military population.

**Methods:**

We compare the effectiveness of the DrinksRation smartphone app with treatment as usual for personnel identified at risk of alcohol-related harm using the Military DrinksRation study that is a 2-arm, single-blind, 1:1 randomized controlled trial of the UK Armed Forces population. It is hypothesized that the DrinksRation app will be more efficacious at reducing alcohol consumption compared to treatment as usual. Recruitment will be predominantly from routine, periodic dental inspections all service personnel regularly undertake, supplemented by recruitment from military-targeted media messaging. The primary outcome is the change in alcohol units consumed per week between baseline and day 84, measured using the timeline follow-back method. Secondary outcome measures are a change in the Alcohol Use Disorders Identification Test score, a change in the quality of life assessment, and a change in drinking motivations and app usability (intervention arm only) between baseline and day 84. A final data collection at 168 days will assess the persistence of any changes over a longer duration.

**Results:**

The study is expected to open in August 2023 and aims to enroll 728 participants to allow for a study sample size requirement of 218 per arm and a 40% attrition rate. It is expected to take up to 12 months to complete. The results will be published in 2024.

**Conclusions:**

The Military DrinksRation study will assess the efficacy of the smartphone app on changing alcohol use behaviors in service personnel. If a positive effect is shown, the UK Defence Medical Services would have an effective, evidence-based tool to use as part of an alcohol management clinical pathway, thereby providing better support for military personnel at risk of harm from alcohol drinking.

**Trial Registration:**

ISRCTN Registry 42646;. https://doi.org/10.1186/ISRCTN14977034

**International Registered Report Identifier (IRRID):**

PRR1-10.2196/49918

## Introduction

### Overview

Consumption of alcohol is highly prevalent in the United Kingdom, with 65% of men and 50% of women reporting having consumed an alcohol-containing drink in the past week [1]. Consumption in the general population has steadily risen since the 1950s, with the most significant increase among women [2,3]. Exploration of drinking trends across the United Kingdom suggests that there has been an overall small reduction in intake in recent years. However, this is due to moderate reductions in the prevalence of drinking (from 70% to 60%) and alcohol intake in young men aged 16-24 years [2]. This reduction masks a 3 unit per week increase in alcohol intake and an increase in the prevalence of drinking by women. While there has been a reduction in the number of drinkers and the amount consumed by the 16-24-year-old age group, they are the group most likely to binge drink, a trend that transfers into the military population. A large cross-sectional study demonstrated that UK military personnel consume alcohol at rates higher than age- and sex-matched civilian personnel [4]. Military personnel are additionally more likely to drink excessively [4,5]. This binge pattern is attributed to the populations being “young single individuals [who] are less likely to have children and other domestic responsibilities that make heavy drinking less frequent or possible” [4] and the military culture of communal risk-taking [5].

Alcohol’s negative effects are wide-ranging and associated with harm to health, accidents, criminality, social disruption, and poor occupational outcomes [1]. For those aged 15-49 years in England, alcohol misuse is the single largest risk factor for ill health and premature death [3]. UK defense data suggest that 59% of military personnel are at risk of alcohol-related harm, which is higher than the general population (42%) [6]. Alcohol was linked to, or the cause of, almost 5% of aeromedical evacuations between 2018 and 2019, and the number of personnel with an alcohol abuse or misuse read code in their primary care clinical notes increased from 457 to 584 between November 2017 and April 2019 [6]. From a health perspective, alcohol is implicated in much of the mental health burden within defense primary health care (DPHC) and complicates other health-related activity through interactions with medication, treatments, and direct exacerbation of symptoms [6,7].

Alcohol is implicated in military disciplinary issues [8,9] and occupationally, alcohol misuse is associated with a loss of productivity, in a dose-dependent manner, through increased rates of sickness absence and presenteeism [3,5]. The occupational impacts of alcohol on the military are more extensive than in the civilian workforce due to the nature of military employment [4,5]. Recognizing the impact that alcohol has on the effectiveness of military personnel, there are various strategies aimed at reducing their alcohol use. Personnel aged 30 years or younger have mandatory alcohol lectures that highlight the harmful effects of excessive consumption and disciplinary issues complicated by alcohol attracting more significant punishment [10].

National guidance recommends alcohol brief interventions (ABIs) for patients identified as consuming alcohol at hazardous or harmful levels [11]. An ABI is “a simple intervention aimed at individuals who are at risk through drinking above the guidelines but are not typically seeking help for an alcohol problem” [12]. All UK Armed Forces (AF) personnel are screened for risky drinking behaviors using the Alcohol Use Disorders Identification Test for Consumption (AUDIT-C) [13] at periodic dental inspections (PDIs). A “brief intervention” is given to personnel who record a score of 4 or more (men) or 3 or more (women). There is concern, however, that recent apparent reductions in alcohol intake reflect reporting bias rather than an actual reduction in the AUDIT-C score. An intentionally low misrepresentation of actual intake would avoid the individual being given an ABI or offered a referral to the general practitioner [14] and avoid any associated concerns about the career impact of being identified as being at risk of harm from drinking.

Existing evidence for the effectiveness of ABIs appears robust, with an extensive Cochrane review determining they can reduce alcohol consumption by 2 standard drinks per week after a year [15]. However, this defined an ABI as “a conversation comprising 5 or fewer sessions of brief advice or brief lifestyle counseling and a total duration of less than 60 minutes” [15]. This exceeds the time available for opportunistic ABIs within DPHC consultations. The Cochrane review also excluded digital interventions, which are intuitively of huge relevance in a young, digital native population such as the AF. Face-to-face interventions may be effective, but not if they are not accessed by a digital generation [16-18]. A narrative review of 10 studies involving military personnel and veterans found that web-based interventions may be of benefit [19]. There remains a paucity of evidence available for the effectiveness of ABIs on military populations, or indeed, for which aspects of behavior change theory are effective in a population with rigid command structures and regulations.

The primary purpose of the DrinksRation randomized controlled trial (RCT) study is to determine whether similar effectiveness is found in a serving military population.

### Military DrinksRation (MDR) Study Aim

DrinksRation is a military-focused smartphone app that has been shown to be effective in reducing alcohol consumption in a veteran, help-seeking population [20]. The primary purpose of this RCT is to determine whether the DrinksRation app has similar effectiveness in a serving, non–help-seeking military population. An effective app could support or integrate into the defense alcohol management pathways and provide support to service personnel while they wait for specialist intervention from defense community mental health services. Additionally, if the links between drinking behaviors and social and health outcomes are better understood, there is scope for future policy design to target the precipitating factors leading to a more positive and healthy life.

## Methods

### Ethical Considerations

This study was approved in September 2023 by the Ministry of Defence Research Ethics Committee (MODREC; registration number: 2154/MODREC/22) and registered as a clinical trial with the International Standard Randomised Controlled Trial (ISRCT) number registry (submission number: 42646). Any amendments to the protocol will be registered with ISRCT and approved by MODREC.

### Study Design

The MDR study is a 2-arm, single-blind, 1:1 RCT of the UK AF population comparing the effectiveness of the DrinksRation smartphone app with treatment as usual (TAU) for those at risk of alcohol-related harm. It is hypothesized that the DrinksRation app will be more efficacious at reducing alcohol consumption compared to TAU.

### Study Sample

A protocol for the veterans’ RCT of the DrinksRation app calculated a sample size of 37 participants in each arm to achieve significance and a target of 620 invitations to cover nonparticipation and attrition [17]. Assessing the sample size for a serving (nonveteran), non–help-seeking population, an effect size of 4 units of alcohol per week, and a mean consumption of 30.5 (SD 14.9) units/week were used. Consumption is based on a large systematic review of face-to-face ABIs [21]. The mean change of 4 units is based on a change of 2 units detected in a systematic review for the general population [21]; 7 units detected in a previous DrinksRation study [16]; and 4 units being used in the help-seeking veteran RCT [17]. The sample size was calculated as 218 per arm. The study will aim to recruit 728 participants to allow for a 40% attrition rate. Defense statistics state that 59% of the serving AF population are at risk of alcohol-related harm [6]. This equates to approximately 7250 personnel per month having a PDI and being eligible for the study.

### Recruitment

Potential participants for the MDR study will be currently serving UK AF personnel identified through PDIs and self-identification following study publicity. All 148,000 serving military personnel are offered PDI at least annually. Before the PDI, all personnel are asked to complete an AUDIT-C screening tool. Those scoring at increasing risk of alcohol-related harm to health (≥5) are given an alcohol brief intervention by the dental staff, which consists of a single brief discussion around the risks of drinking lasting 2-3 minutes. Those scoring in the higher risk group (8-10) or suggesting possible dependence (11-12) are offered a referral from the dentist to a general practitioner for follow-up.

The trial will be publicized through service-specific communications, social media, and recruitment posters displayed across the UK defense estate. Additionally, potential participants will be identified through service-specific communication, including links and QR codes to the study website promoted through single service information pathways and on the defense intranet. MyNavy and similar apps within the defense gateway will also be used to announce the trial and direct potential participants to the study website. Military-focused social media, such as #MilTwitter and AF-specific Facebook groups, will be asked to promote the study and host links to the study website. Posters with a short link and QR code for the study website will also be displayed in areas of high footfall in large establishments.

All potential participants will be screened for eligibility using a web-based AUDIT-C tool [13] on Research Electronic Data Capture (REDCap) [22]. Those scoring ≤4 will not be eligible for the DrinksRation RCT. Personnel scoring ≥5 will be given an ABI either in the form of the standard defense guidance on reducing alcohol use or by the dental staff as per usual management. Personnel will then be directed to the participant information sheet (PIS), eligibility criteria, and web-based consent form on REDCap.

Participants will be included in the study if they are currently serving UK AF; have an Android or iOS smartphone and are willing or able to download the DrinksRation app; report an AUDIT-C score of ≥5; and provide a telephone number or email address for participant safety purposes.

Participants will be excluded if they are unwilling or unable to download the DrinksRation app or if they scored ≤4 on AUDIT-C. There will be no exclusion on the grounds of harmful drinking identified by AUDIT-C screening.

There are no age or sex restrictions on participation beyond those of the military population. Specifically, personnel aged 18 years or younger are not excluded from the MDR study, as they form a small proportion of the UK military population. While the purchase of alcohol by those aged 18 years or younger is illegal, consumption by underage personnel does happen, and age does not proffer any immunity against potentially harmful drinking behaviors [23]. Indeed, living with people who are drinking alcohol increases the likelihood of a child drinking (odds ratio 3.6 for children living with 3 or more drinkers) [3].

After consenting to inclusion in the study and the collection of baseline demographics, participants will be randomized to the intervention group or TAU group using the embedded REDCap randomization module.

### Intervention

**Figure 1:**
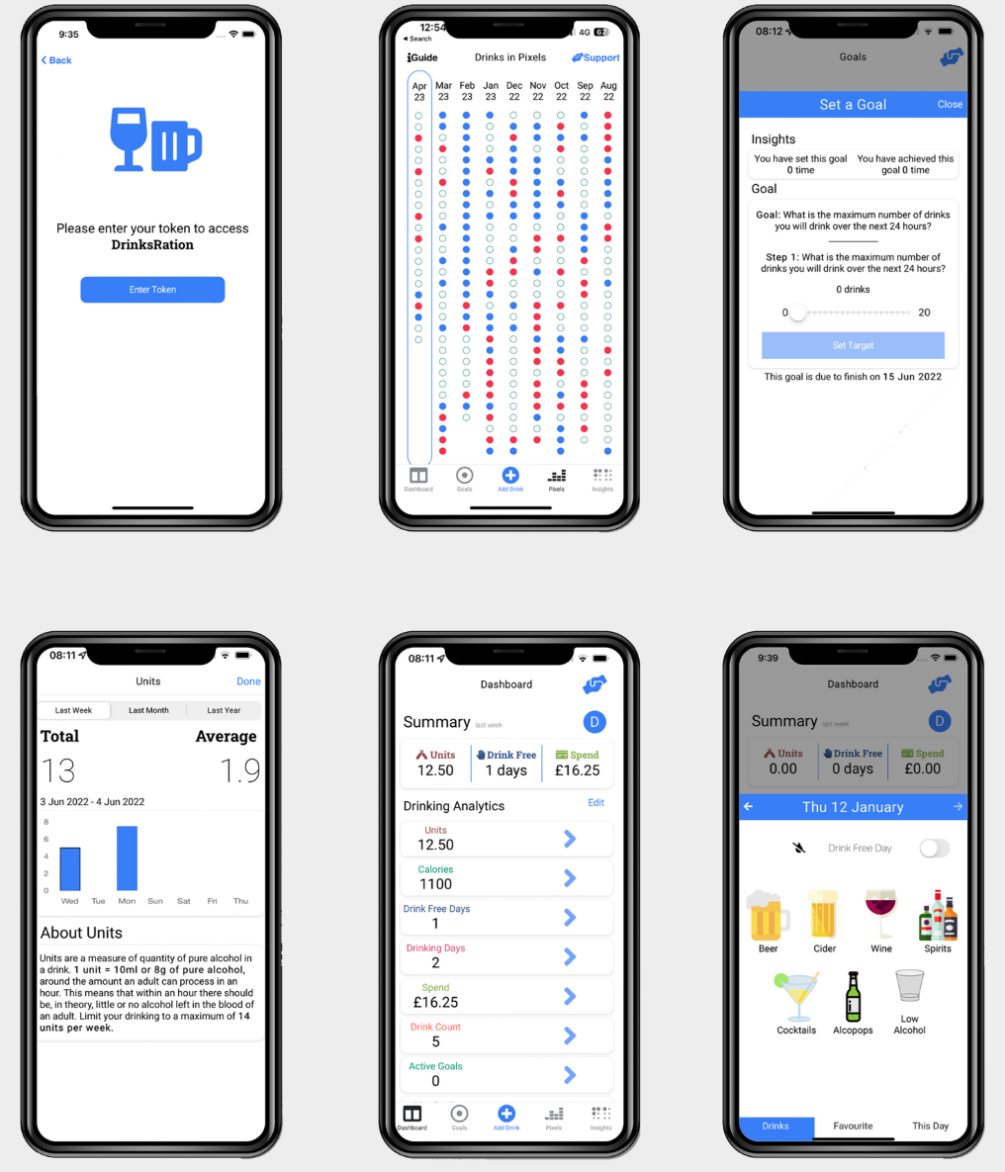
Military DrinksRation randomized controlled trial: smartphone app screenshots.

**Figure 2:**
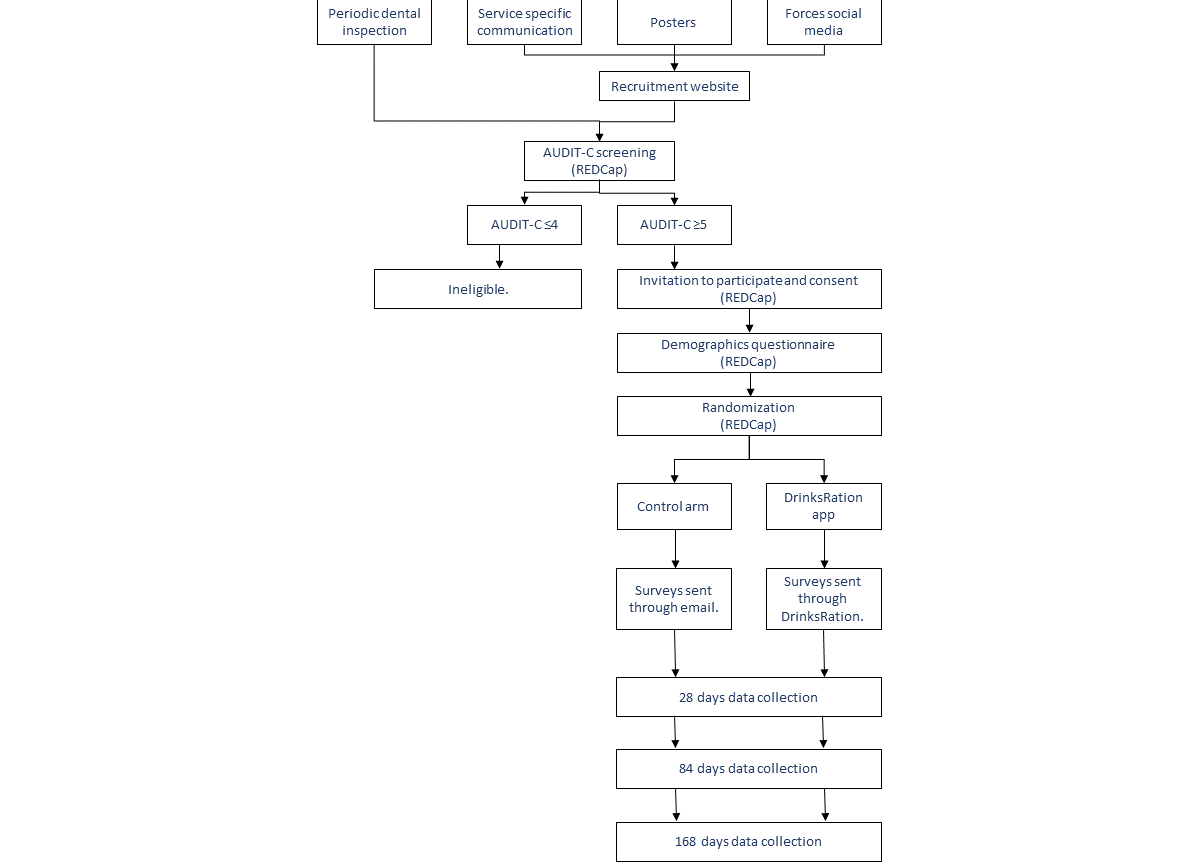
Study flow diagram. Primary and secondary outcomes are assessed at day 84, with outcomes reassessed at day 168 in order to understand the longer-term benefits of the intervention. AUDIT-C: Alcohol Use Disorders Identification Test for Consumption; REDCap: Research Electronic Data Capture.

Participants randomized to the intervention arm of the RCT will be sent an email link to the DrinksRation app with a unique reference code to enable data linkage between REDCap and DrinksRation. The app’s function, theoretical basis, and supporting evidence are fully described elsewhere [16,20,24]. Images of the app and its functionality are shown in Figure 1. After downloading the app, on first use, participants will be prompted to complete baseline assessment surveys. App usage and further surveys were then presented by the app with push notifications to the participant, highlighting the need for completion. Participants will be asked to use the app for a minimum of 28 days. Participant flow through the study is shown in Figure 2.

### Control

Participants randomized to the control arm will receive baseline assessment and all further surveys through email with the same periodicity as the intervention group. The control group will be asked to complete the surveys and will be given access to the DrinksRation app at the end of the study period. Noncompletion of surveys will prompt an automated push notification (intervention arm) or email (control arm) reminder after 24 hours. The structural difference between the arms was considered unavoidable due to the difficulty of creating a sham digital psychological intervention [25]. If a pared-down “dumb” version of the app were offered to the control arm, then it would be difficult to determine whether any effect was due to the visibility of the app or interaction with the app. A previous study of the DrinksRation app showed a large attrition rate in the control arm when participants were given a dumb version of the app [20].

### Randomization and Blinding

Participants will be randomized using the in-built randomization module of the REDCap program. This module automatically allocates participants to either the DrinksRation app or the TAU group based on a randomization table generated on the internet using the Sealed Envelope website [26]. Two participant blocks in a 1:1 ratio will be used. Randomization will be stratified by sex because it has previously been shown that alcohol use in the UK military is different between the sexes [4]. The randomization module will be programmed by the study independent medical officer (IMO), who is not blinded to the allocations in order to enact the safety protocols if required. The researchers had no visibility of programming, and role-based access to REDCap prevented access to any identifiable information or allocation for the research team. There was no contact between researchers and participants at any stage of the trial.

Participants were blinded to the intervention as they were told that the research involved them completing various surveys about drinking behaviors, health, and well-being. The study IMO will not be blinded to allocation and will have data access rights allowing him to link the participant reference codes to personal identifiers in case of any adverse events.

### Measures

The primary outcome measure is a change in alcohol units consumed per week between baseline and day 84, as measured by the timeline follow-back method [27]. Secondary outcomes are a change in the Alcohol Use Disorders Identification Test (AUDIT) score, a change in the quality of life assessment, and a change in drinking motivations and app useability. While the DrinksRation intervention aims to reduce alcohol intake at 84 days, a final data collection at 168 days will assess any persistent benefits.

Data will be collected through a selection of surveys pushed to the participants in line with the periodicity detailed in Table 1.

The study instruments are all validated for the general population and are detailed in Table 1. The alcohol and mental health symptomatology surveys were included in the veteran’s trial. The MDR trial additionally includes surveys for drinking motivations (Drinking Motives Questionnaire 9-item tool [DMQ-R]), recent life events (Recent Life Events Questionnaire [RLE-Q]), gambling motives (Gambling Motivations Questionnaire [GMQ-9]), and domestic abuse and sexual assault (National Crime Survey for England and Wales). These behaviors have been associated with both alcohol consumption [3] and a military population [40,41]. A tertiary outcome of the MDR study is to assess the correlation of these issues with alcohol use within the military population. This information does not contribute to the assessment of the app’s effectiveness, but it is valuable information for UK defense policy makers.

**Table 1:** Summary of measures and data collection time points.

Measure	Total questions	Day 0	Day 0-1	Day 3	Day 7	Day 28	Day 84	Day 168
Demographics	18	✓						
AUDIT-C^a^ [13]	3	✓						
Participation information and consent	N/A^b^	✓						
Alcohol use (AUDIT^c^) [28]	10		✓			✓	✓	✓
Alcohol intake (TLFB^d^) [27]	N/A		✓			✓	✓	✓
Resource allocation (YAACQ^e^) [29]	24		✓				✓	✓
Readiness to change ruler	1		✓					
Self-efficacy ruler	1		✓					
Depression (PHQ-2^f^) [30]	2		✓			✓		
Anxiety (GAD-2^g^) [31]	2		✓			✓		
International trauma questionnaire for PTSD^h^ (PC-PTSD-5^i^) [32]	5		✓			✓		
World Health Organization Quality of Life-BREF^j^ (WHOQOL-BREF) [33]	27			✓		✓	✓	✓
Loneliness (De Jong Gierveld) [34]	11			✓		✓		
Drinking motivations (DMQ-R^k^) [35]^l^	20			✓			✓	
Recent life events (RLE-Q^m^) [36]^l^	21			✓				
Gambling motives (GMQ-9^n^) [37]^l^	9				✓			
Domestic abuse (NCSE&W^o^) [38]^l^	30				✓			
Sexual assault (NCSE&W) [38]^l^	13				✓			
mHealth App Usability Questionnaire (MAUQ) [39]	16					✓		
Approximate time to answer (min)^p^	—^q^	10	13	15	9	20	15	15

^a^AUDIT-C: Alcohol Use Disorders Identification Test for Consumption.

^b^N/A: not applicable.

^c^AUDIT: Alcohol Use Disorders Identification Test.

^d^TLFB: timeline follow-back.

^e^YAACQ: Young Adult Alcohol Consequences Questionnaire.

^f^PHQ-2: Patient Health Questionnaire 2-item tool.

^g^GAD-2: Generalized Anxiety Disorder 2-item tool.

^h^PTSD: posttraumatic stress disorder.

^i^PC-PTSD-5: Primary Care-Posttraumatic Stress Disorder 5-item screening tool.

^j^BREF: Best Available Technique Reference Document.

^k^DMQ-R: Drinking Motives Questionnaire 9-item tool.

^l^Not included in veterans’ DrinksRation study.

^m^RLE-Q: Recent Life Events Questionnaire.

^n^GMQ-9: Gambling Motivations Questionnaire.

^o^NCSE&W: National Crime Survey for England and Wales.

^p^Allowing 10 seconds per question, rounded up and with additional comfort time added.

^q^Not available.

### Statistical and Analysis Plan

The MDR study is based on a recently completed study of veterans, which was sponsored by the Forces in Mind Trust. Analysis will therefore be undertaken in accordance with the published protocol for that study [16,20]. There will be no interim analysis. The threshold for significance will be *P*=.05. Effect sizes will be reported.

Descriptive statistics (eg, demographics and response rate) and independent sample *t* tests and chi-square tests will be carried out to explore and identify potential differences between the intervention and control arms at follow-up. An intention-to-treat method will be used for primary outcome analysis, such that those who are lost to follow-up will be retained in the primary analysis. Multiple imputations will be performed to estimate missing data, where appropriate. The primary outcome analysis will examine whether there is a statistically significant difference between the intervention and control arms on change in self-reported timeline follow-back UK units consumed. Repeated-measures mixed modeling analyses will be conducted to examine the primary hypothesis that those randomized to receive the DrinksRation app will report a greater reduction in alcohol consumption compared with control participants from baseline to 3-month follow-up (day 84).

For the secondary outcomes, changes in the AUDIT score and World Health Organization Quality of Life- Best Available Technique Reference Document (WHOQOL-BREF) computed quality of adjusted life years will be assessed using repeated-measures mixed modeling. These analyses will be repeated to assess changes between baseline and follow-up and will serve as a secondary outcome to assess the long-term impact of the intervention on participants [17]. Tertiary outcomes will be assessed for correlation with alcohol consumption and will be reported separately to the RCT outcome.

The study will be reported in line with the CONSORT (Consolidated Standards of Reporting Trials) [42] criteria for RCTs and the Template for Intervention Description and Replication guide [43]. The study was designed in accordance with the Standard Protocol Items: Recommendations for Interventional Trials (SPIRIT) statement [44].

### Data Management and Confidentiality

All study data will be captured and held electronically within REDCap or the DrinksRation app, depending on the participant arm. There is no physical paperwork and no requirement for any. Consent forms are held electronically within REDCap. Data will be processed and stored in line with the General Data Protection Regulation (GDPR) Article 6 (1.e) “processing is necessary for the performance of a task carried out in the public interest or in the exercise of official authority vested in the controller” [45]. DrinksRation app data are stored on Google-managed, UK-based servers contracted by the King’s Centre for Military Health Research (KCMHR). All other study data are held within the defense medical services REDCap servers at the University of Birmingham.

### Data Monitoring

A data monitoring committee is not necessary because the intervention is well-defined and not considered to be a risk to participants [46]. Prompts to complete surveys will be sent to participants through the app (intervention arm) or by email (control arm). A reminder notification will be sent if surveys are not completed within 24 hours. There is no further follow-up for missing data. Missing data do not impact further survey prompts being sent; all participants will receive notification when the next survey is due in accordance with Table 1. No additional plan to promote participant retention is required because the app evaluation needs to consider natural attrition rates and adherence to app usage.

### Adverse Events

The investigators will review the data weekly to identify any potential adverse events. As per the veteran’s DrinksRation study, an adverse event is defined as a participant either consuming more than 25 units of alcohol in a 24-hour period or giving survey responses suggesting a significant mental health problem [17]. Additionally, participants can register an adverse event or problem by contacting the researchers through the study email address or website.

**Figure 3:**
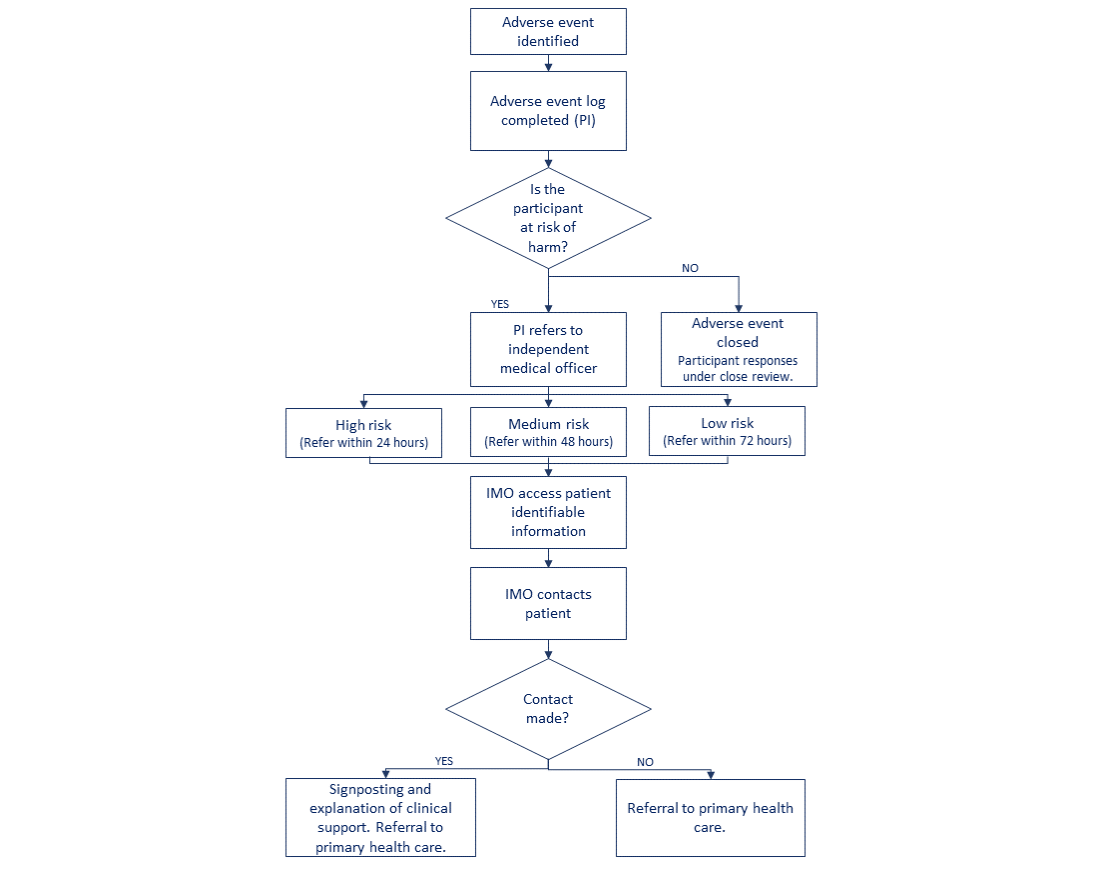
Adverse events management plan showing the unblinded role of the independent medical officer (IMO) in order to address potential harms. PI: principal investigator.

If an adverse event is identified, the principal investigator (PI) will review the available data for the participant, and if there are concerns, the PI will inform the IMO. The IMO will have access to the patient identifiable information and will contact the patient to facilitate referral to primary care if required and assess suitability to continue in the trial. Figure 3 details the adverse event management plan. All adverse events will be recorded in a risk event log held within the REDCap research database.

### Study Consent and Withdrawal

Consent will be managed on the internet through REDCap; immediately after AUDIT-C screening, potential participants are shown the PIS and study consent form. Consent is captured electronically within the REDCap platform. Participants who want time to think about participation or to ask questions can do so by using the option to “save and return later,” therefore allowing participants to consider their participation at their leisure. The study website hosts a “frequently asked questions” document and a contact email address for the PI. Staff in dental centers will be given a standard briefing about the study and asked to direct further questions to the investigators through the study email address. Participants will be informed that their data will be used primarily for the purposes of the MDR study. However, if results indicate that further studies are beneficial for the military community, the data may be further processed to extend the scope of the research in the field of alcohol behavior change. If this is deemed beneficial, then participants will be informed before the compatible processing occurs.

No payment for participation will be made to ensure that payment does not impact app usage rates. Any participant who sustains harm as a direct result of the study can apply for compensation under the Ministry of Defence’s No-Fault Compensation Scheme. Details of the scheme will be available to participants with the PIS. The DrinksRation app has been designed and built by KCMHR, is published with open-source coding, and is free to download. It does not provide financial benefit. The PI is employed by the Royal Navy, and academic fees associated with this study are paid for by defense; however, defense has had no influence over the content or design of this trial.

All the data gathered will be written up as part of the thesis submitted for an MD (Research) in Psychological Medicine Research at King’s College London. Results will be published with open access in line with best practices [47]. Authorship will be as per this paper. Additionally, an “executive summary” will be written to facilitate access to findings by nonresearchers across defense. Participants wanting to be informed of the study outcomes will be emailed the summary and a link to any published article. Findings will be fed back to key stakeholders within defense.

**Figure 4:**
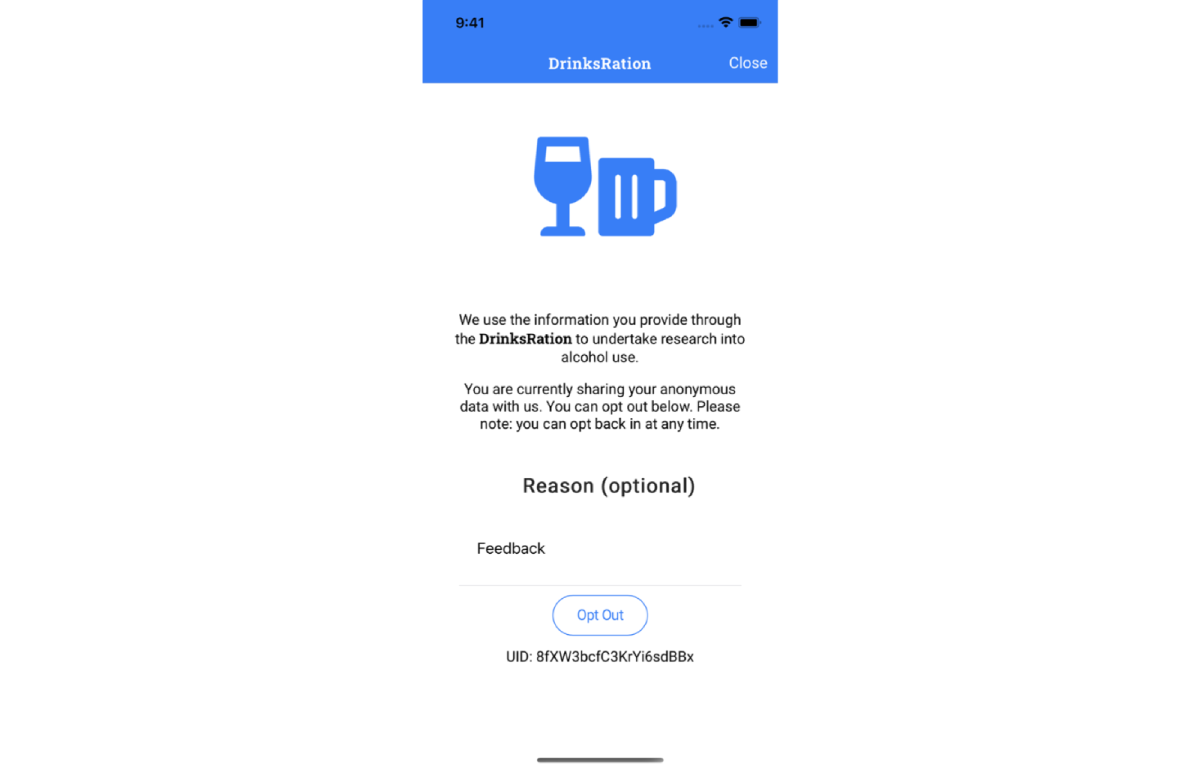
Participant withdrawal screen, which includes the ability to delete the account or withdraw from the study.

Participants are able to withdraw from the study at any point up to the point when the data capture is extracted for analysis. At this point, the data will be grouped, and it will no longer be possible to select individual data. Withdrawal from the intervention arm is managed entirely from within the DrinksRation app. Participants visit the “Settings” tab, and the option to withdraw is visible (see Figure 4 below). To withdraw from the control arm, participants will need to email the PI, either directly or through the study website. Withdrawing participants are given the option to delete the data already collected or allow the research team to use this data.

## Results

Recruitment will commence in August 2023 and be completed by October 2023. Results are expected by autumn 2023.

## Discussion

The existing evidence for brief interventions in alcohol behavior management is robust, with several large systematic reviews published in this area [15,19,21,48,49]. The types of brief interventions used in these reviews are varied and often resource-intensive, with many of the included interventions not aligning with the National Institute of Health and Care Excellence guidelines. These face-to-face interventions have a place in an evidence-based alcohol management strategy but come with a training burden and a requirement for a significant uplift in primary care appointments in order to address the high levels of potentially hazardous drinking in the AF population. This considerably limits their practicality because they exceed the time available within standard primary health care appointments of 10-15 minutes. Digital interventions have been developed to address this issue and have also been shown to have an effect [50]. There are 2 systematic reviews of digital alcohol interventions identified on NHS Evidence [50,51]. They report that digital interventions can reduce alcohol consumption. The larger Cochrane review reports that brief interventions are responsible for alcohol reductions of 22.8 (95% CI 15.4-30.3) g of alcohol per week compared to no intervention [50], approximately 2 standard drinks per week. The smaller review included observational and experimental study types and so quantitative synthesis was not undertaken. A digital solution may be desirable to a digital native population [18] and confers resource advantages over face-to-face interventions. Fundamentally, face-to-face interventions may be effective, but they also may not be accessed by the digital generation, which forms the majority of the current serving AF population [16-18].

The UK AF population also has a relationship with alcohol that differs from that of the general public [5], and there is no existing evidence for the use of brief interventions within the UK AF. A single systematic review including UK AF personnel and veterans was found, but the heterogeneity and risk of bias within the included studies precluded a meta-analysis. The data and narrative synthesis showed that digital interventions may be effective in this mixed population [19]. Interestingly, a statistically significant reduction in binge drinking (mean difference –0.24, 95% CI –0.35 to –0.13 binges/week) [50] was seen with digital interventions, which is not seen in face-to-face interventions (mean difference –0.08, 95% CI –0.28 to 0.12 binges/week) [21]. The finding of effect in terms of binge drinking also makes digital interventions especially beneficial to the AF population [52,53].

The DrinksRation app fulfills the requirement for the intervention to be delivered within available time resources in DPHC and has been shown to be effective in a small-scale study of veterans [16,54]. However, there are some significant demographic differences between the serving and veteran populations, namely the age profile. A third of veterans are aged 80 years or older; half are aged 65 years or older; and less than 10% are aged 30 years or younger [55]; compared to just under half of the serving AF population being 30 years old or younger [56]. This study will determine whether the DrinksRation app is effective at changing the alcohol use behavior in this younger, serving AF population and, for the first time, provide defense medical services with an evidence-based alcohol intervention designed for military personnel.

## Appendix

Multimedia Appendix 1 Defence Medical Services Research Steering Group- Endorsement Letter-Dec 20.

Multimedia Appendix 2 Peer Review comments from Defence Medical Services Research Steering Group Sep 20.

## Abbreviations

ABI: alcohol brief intervention
AF: Armed Forces
AUDIT: Alcohol Use Disorders Identification Test
AUDIT-C: Alcohol Use Disorders Identification Test for Consumption
CONSORT: Consolidated Standards of Reporting Trials
DMQ-R: Drinking Motives Questionnaire 9-item tool
DPHC: defense primary health care
GDPR: General Data Protection Regulation
GMQ-9: Gambling Motivations Questionnaire
IMO: independent medical officer
ISRCT: International Standard Randomised Controlled Trial
KCMHR: King’s Centre for Military Health Research
MDR: Military DrinksRation
MODREC: Ministry of Defence Research Ethics Committee
PDI: periodic dental inspection
PI: principal investigator
PIS: participant information sheet
RCT: randomized controlled trial
REDCap: Research Electronic Data Capture
RLE-Q: Recent Life Events Questionnaire
SPIRIT: Standard Protocol Items: Recommendations for Interventional Trials
TAU: treatment as usual
WHOQOL-BREF: World Health Organization Quality of Life- Best Available Technique Reference Document

## Data Availability

The data sets generated during this study are not publicly available in line with Ministry of Defence research policies but are available from the corresponding author on reasonable request.
