# Supplementary material for: The DrinksRation Smartphone App for Modifying Alcohol Use Behaviors in UK Military Service Personnel at Risk of Alcohol-Related Harm: Protocol for a Randomized Controlled Trial
Source: JMIR Res Protoc. 2023 Oct 13;12:e49918. doi: 10.2196/49918 (PMC10612007; doi:10.2196/49918)
Supplement: Multimedia Appendix 1 [file resprot_v12i1e49918_app1.pdf]

Strategic Command
Headquarters Defence Medical Services Group

Samantha Brown
Research Business Manager
HQ Defence Medical Services
ICT Centre, Birmingham Research Park
Birmingham
B15 2SQ
Telephone +44 (0)121 415 8903
Email: Samantha.Brown275@mod.gov.uk

FAO: Surg Cdr Kate King
Col Mike Smith

16 December 2020

Dear Kate,

Re: An evidence based approach to alcohol brief intervention with Defence Primary Health Care

Many thanks for your presenting your proposal to the DMSRSG today.

Without exception, the group found your presentation informative and were confident in your methodology.

They all considered your proposal to be of significant benefit to the MOD and are happy to endorse your application.

Best Wishes

Yours sincerely,

Sam

Samantha Brown
Research Business Manager
