# Supplementary material for: The DrinksRation Smartphone App for Modifying Alcohol Use Behaviors in UK Military Service Personnel at Risk of Alcohol-Related Harm: Protocol for a Randomized Controlled Trial
Source: JMIR Res Protoc. 2023 Oct 13;12:e49918. doi: 10.2196/49918 (PMC10612007; doi:10.2196/49918)
Supplement: Multimedia Appendix 2 [file resprot_v12i1e49918_app2.pdf]

From:Amarjit Samra, Director of Research

Director of Research
Medical Directorate
HQ Defence Medical Services
ICT Centre
Birmingham Research Park
Birmingham.
B15 2SQ

Telephone: +44 (0)121 415 8861
E-mail: Amarjit.samra938@mod.gov.uk

22nd September, 2020

Dear Kate,

I would like to thank you for submitting your research proposal on ‘An evidence based approach to alcohol brief intervention within Defence Primary Health Care’ to the DMSRSG. Your presentation on 16 September was informative and all members of research steering group, recognised the need for research into this important area for the military.

As you are aware that this group considers the scientific robustness of the study, the members’ thought that there was insufficient information in your proposal and presentation on the actual study. The outcome of the discussion was that you need to include the following information into your proposal when you resubmit: Primary outcome measures, sample size, what is going to be your recruit process, who is going to be recruited, from where will these participants be recruited?

You have the opportunity to address the above issues and present your proposal at the next Defence Academic Working Group meeting scheduled for 16 December or at the next DMSRSG scheduled for 18 March 2021.

I am sure this is disappointing news for you, but let me reassure that the committee considers this is to be an important area and with a more robust proposal, I am sure that it will be approved.

Kind regards,

Amarjit
